# Amount and composition of total fatty acids in red and yellow bone marrow are altered with changes in bone mineral density

**DOI:** 10.17179/excli2023-5843

**Published:** 2023-02-13

**Authors:** Sabrina Ehnert, Anna J. Schreiner, Claudine Seeliger, Josef Ecker, Fabian Springer, Gerhard Liebisch, Philipp Hemmann, Tina Histing, Andreas K. Nussler

**Affiliations:** 1 Siegfried-Weller Institute for Trauma Research, BG Trauma Center, University of Tuebingen, Tuebingen, Germany; 2 RKH Orthopedic Clinic Markgroeningen, 71706 Markgroeningen, Germany; 3 ZIEL Institute for Food & Health, Research Group Lipid Metabolism, Technical University of Munich, Freising, Germany; 4 Department of Diagnostic and Interventional Radiology, University Hospital and BG Trauma Center, University of Tuebingen, Tuebingen, Germany; 5 Institute of Clinical Chemistry and Laboratory Medicine, University Hospital Regensburg, Regensburg, Germany

**Keywords:** bone mineral density, fatty acid composition, bone marrow, saturated fatty acids, unsaturated fatty acids, Tartrate-Resistant Acidic Phosphatase (TRAP5b)

## Abstract

There is general consent that with decreasing bone mineral density the amount of marrow adipose tissue increases. While image-based techniques, claim an increase in saturated fatty acids responsible for this effect, this study shows an increase in both saturated and unsaturated fatty acids in the bone marrow. Using fatty acid methyl ester gas chromatography-mass spectrometry, characteristic fatty acid patterns for patients with normal BMD (N = 9), osteopenia (N = 12), and osteoporosis (N = 9) have been identified, which differ between plasma, red bone marrow and yellow bone marrow. Selected fatty acids, *e.g.* FA10:0, FA14:1, or FA16:1 n-7 in the bone marrow or FA18:0, FA18:1 n-9, FA18:1 n-7, FA20:0, FA20:1 n-9, or FA20:3 n-6 in the plasma, correlated with osteoclast activity, suggesting a possible mechanism how these fatty acids may interfere with BMD. Although several fatty acids correlated well with the osteoclast activity and BMD, there was not a single fatty acid contained in our fatty acid profile that can be claimed for controlling BMD, a fact that may be attributed to the genetic heterogeneity of the patients.

## Introduction

There is global evidence that life span and overall health improves (Tuljapurkar et al., 2000[30]). However, the steadily aging population and increasingly sedentary lifestyles have made low bone mass, *i.e.* osteopenia and osteoporosis, and associated fragility-fractures a serious public health concern. Despite a minor trauma, fragility-fractures frequently result in work absence, decreased productivity, disability, deterioration of health, and reduced quality of life.

Directly after birth, the bone marrow is comprised mainly of red bone marrow (RBM), which contains its red color from large amounts of hematopoietic, osteogenic, and erythroid cells. With increasing age, the bone marrow adipose tissue (MAT) steadily expands, resulting in the development of yellow bone marrow (YBM) (Moerman et al., 2004[22]). Overall this development is comparable between mice and humans, however, in mice significant strain differences exist. C57BL/6 mice, which have an overall low BMD, have very low numbers of marrow adipocytes in their long bones. In contrast, the long bones of C3H/HeJ mice (a distinct strain), which have a very high BMD, contain a lot more marrow adipocytes (Beamer et al., 1996[1]). This challenges a causal relationship between simply the amount of MAT and the BMD. Instead, factors, *e.g.* fatty acid composition and genetic variability in lipid metabolism get into focus. Picking up the previous example, C3H/HeJ mice have been shown to have higher levels of triglycerides and cholesterol in their plasma than C57BL/6 mice (Jiao et al., 1990[14]), suggesting that not only the fatty acid composition could be relevant, but also that the fatty acid composition in the plasma and the MAT correlate.

However, one of the unsolved key questions about MAT and bone is whether accumulation of MAT precedes, parallels, or follows bone loss. MAT comprises a large proportion of the marrow cavity in primary and secondary osteoporosis (Cohen et al., 2012[4]; Devlin and Rosen, 2015[6]). A study using proton magnetic resonance spectroscopy proposed that saturated lipids increase preferentially to unsaturated lipids in marrow fat of patients with decreased BMD (Yeung et al., 2005[31]). Therefore, it was proposed, that nutritional modification of the fatty acid composition may improve the BMD (Martyniak et al., 2021[20]). While the protective role of omega-3 poly-unsaturated fatty acids (PUFA) in pathological calcifications, *e.g.* atherosclerosis, is indisputable, their effects on BMD is less clear. Although a diet rich in omega-3 PUFA could improve BMD in different rodent models, comparable studies in humans showed contradictory results - for review see (Sharma and Mandal, 2020[28]). More detailed analyses of the fatty acid composition in the bone marrow using gas chromatography (Griffith et al., 2009[10]; Miranda et al., 2016[21]), challenged the initial assumption that especially saturated fatty acids (SAT) are increased in the bone marrow of osteoporotic patients (Yeung et al., 2005[31]).

Based on the mentioned literature it is assumed that: (i) with decreasing BMD, the overall amount of fatty acids increases in the bone, (ii) the fatty acid composition in osteoporotic bone is altered, (iii) the fatty acid composition of the well-perfused red bone marrow correlates with the fatty acid composition of both the plasma and yellow bone marrow, (iv) and these alterations in the fatty acid composition in the plasma and bone marrow can be used to screen for osteopenia and osteoporosis.

To investigate these hypotheses, it is planned to characterize the fatty acid composition in the circulating blood (plasma - prior to surgery), the well-perfused red bone marrow (RBM), and the yellow bone marrow (YBM). As the literature suggests that the donor site might affect the fatty acid composition (Griffith et al., 2009[10]), only explanted femur heads will be used to obtain the RBM and YBM. The total fatty acid composition (12 SAT, 7 MUFA, and 11 PUFA) in the plasma, RBM and YBM will be quantified using gas chromatography-coupled to mass spectrometry (GC-MS) and associated to the age-adjusted BMD (T-scores), and circulating bone markers to identify disease-specific pattern.

## Material and Methods

### Ethics statement

The study includes patient material and was performed in accordance with the Declaration of Helsinki (1964) in its latest amendment. Clinical data, blood, and bone-marrow samples from a total of 30 patients receiving a planned primary total hip arthroplasty were obtained in accordance with the ethical vote 228/2017BO2 (approved: 24.05.2017). All study participants have signed written informed consent. Patients below 18 years of age, not capable of consent, or with viral or bacterial infection, were excluded from the study.

### Patients' samples

#### Red and yellow bone marrow

Yellow and red bone marrow were obtained from a total of 30 patients undergoing a total hip arthroplasty at a level 1 trauma center, immediately after resection of the femoral heads. Samples were stored at -80 °C until further use. The associated age-adjusted BMD (T-scores) were determined by quantitative computer tomography (qCT) scans with a reference block (Phantom EFP-06-96).

#### Blood sampling

From the same patients 3.7 mL blood (EDTA plasma) was obtained during a routine blood sampling before the surgery. Blood samples were centrifuged at 1,000 g for 10 min at room temperature 30 min after sampling, to allow clotting. Serum samples were stored (aliquots) at -80 °C until further use.

### Enzyme-linked Immunosorbent Assay (ELISA)

Target proteins in plasma samples were quantified with the help of ELISA kits, performed as indicated by the manufacturers: Bone alkaline phosphatase (Ostase^®^ BAP / AC-57DF1, IDS, Tyne & Wear, UK) and tartrate-resistant acidic phosphatase (TRAP5b / SB-TR201A, IDS) were detected as activity markers for osteoblast and osteoclast, respectively. Cross-linked C-telopeptides of Type 1 collagen (CICP / 1:12.5 sample dilution / #8003 / TecoMedical, Sissach, CHE) and C-terminal telopeptide of fibrillar collagens (CTX-I / 1:3 sample dilution / AC-57SF1, IDS) were detected as markers for collagen formation and degradation. Furthermore, the fat-soluble 25(OH) vitamin D_3 _(25(OH)D_3_ or calcidiol / AC-57DF1, IDS) was detected.

### Lipidomics analysis

Total fatty acid composition was determined based on fatty acid methyl ester gas chromatography-mass spectrometry (FAME GC-MS) in samples from plasma, red and yellow bone marrow, N = 30 respectively (Ecker et al., 2012[8]). 10 to 20 mg of bone marrow tissue and 100 µL plasma were snap-frozen on dry ice immediately after excision and stored at -80 °C until extraction. Tissue samples were weighed in tubes with 700 mg lysing matrix D (#116913050-CF, MP biomedicals, Lake Forest, CA, USA) and allowed to thaw on wet ice. The concentration was set to 0.05 mg/μL using equal parts MeOH and water (#1060181000, Merck, Darmstadt, GER). Tissues were lysed using a homogenizer (FastPrep) set to 30 s and 6 m/s. Transesterification of 10 µL serum and 1 mg bone marrow in solution was carried out as previously described and FAMEs were extracted using hexane (#1007951000, Merck) (Lepage and Roy, 1986[18]; Ecker et al., 2012[8]). GC-MS based total FA analysis was performed as previously published (Ecker 2012[8]). FA13:0 iso as well as FA21:0 iso were used as internal standard for quantification. Total fatty acid amount is presented as nmol/mg protein. Individual fatty acid compositions are presented as molar percentages of the total fatty acid profile. The analyzed fatty acid profile is summarized in Table 1(Tab. 1).

### Statistics

The numbers of patients/donors (N) and technical replicates (n) for each experiment are given in the figure legends. Multivariate analyses including correlation matrices and partitioning were done with JMP 16.0.0 (SAS Institute GmbH, Heidelberg, GER). Individual data points are displayed in scatter plots. Correlations between two factors are displayed as bivariate fitted normal ellipses (*p* = 0.90) and summarized as colored significance circles. Data comparing groups are summarized as box plots with individual measurement points - each with median and interquartile range - analyzed and visualized with GraphPad Prism Version 8 (Dotmatics, Boston - MA, USA). Due to the sample size, a Gaussian distribution could not be assumed and data were compared by non-parametric Kruskal-Wallis test with Dunn's correction for multiple comparisons. A *p *< 0.05 was considered significant.

## Results

### The amount of total fatty acids increased in the bone marrow with low BMD

**Figure 1:**
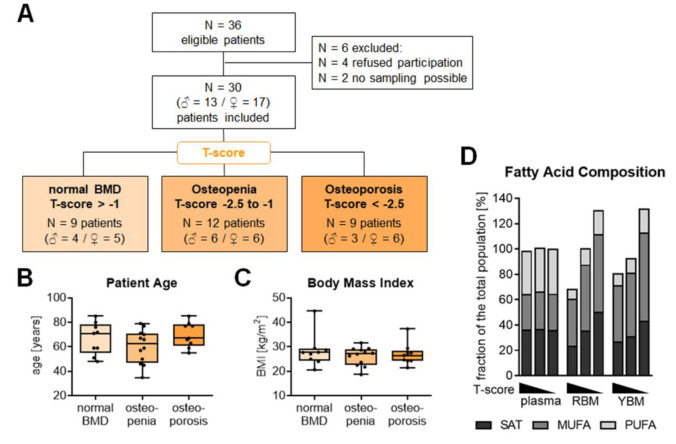
Overview of the patients analyzed in this study. (A) CONSORT diagram summarizing the patients included in this study. N = 30 patients were included in the study, which got grouped into three groups based on their age-adjusted bone mineral density (T-score). Summary of the (B) age and (C) body mass index (BMI) of the included patients. Data are summarized as box plots with individual data points. Groups were compared with non-parametric Kruskal-Wallis test, followed by Dunn's multiple comparison test. Between the three groups, no significant (*p* < 0.05) difference in the patients' age and BMI was detected. (D) The composition of saturated fatty acids (SAT), mono-unsaturated fatty acids (MUFA), and poly-unsaturated fatty acids (PUFA) in the plasma, red bone marrow (RBM), and yellow bone marrow (YBM) is summarized as stacked bars. The total amount of fatty acids is presented as % of the average amount of all 30 samples investigated.

Between July 2017 and March 2018 patients were recruited for this study. From 36 eligible patients 6 had to be excluded from the study, because the patients refused study participation (N = 4) or sampling failed (N = 2). The 30 patients included in this study were then grouped based on their age-adjusted BMD (T-score). 9 patients (4 males and 5 females) had a T-score above -1.0 representing a normal BMD. 12 patients (6 males and 6 females) had a T-score between -1.0 and -2.5, characteristic for osteopenia. The remaining 9 patients (3 males and 6 females) had a T-score below -2.5, characteristic for osteoporosis (Figure 1A(Fig. 1)). The average age and body mass index were comparable between the three groups (Figure 1B, C(Fig. 1)). The amount of total fatty acids and their composition in the plasma were comparable between the three groups. On average, the amount of saturated fatty acids (SAT) was comparable between plasma, red bone marrow (RBM), and yellow bone marrow (YBM). The amount of mono-unsaturated fatty acids (MUFA) in the plasma was approx. half of that in the RBM or YBM. In contrast, the plasma contained approx. 2.5-fold more poly-unsaturated fatty acids (PUFA) than the RBM or YBM. The overall amount of fatty acids in the bone marrow increased with decreasing T-score. The effect was most pronounced in the YBM, where patients with osteoporosis had almost double the amount of fatty acids than patients with normal BMD. The amount of SAT increased by 61.5 % in the RBM and 73.3 % in the YBM. The amount of MUFA increased by 56.2 % in the RBM and 66.2 % in the YBM. The amount of PUFA increased by 101.1 % in the RBM and 146.7 % in the YBM (Figure 1D(Fig. 1)).

### The amount of saturated fatty acids increases in the bone marrow with decreasing BMD

**Figure 2:**
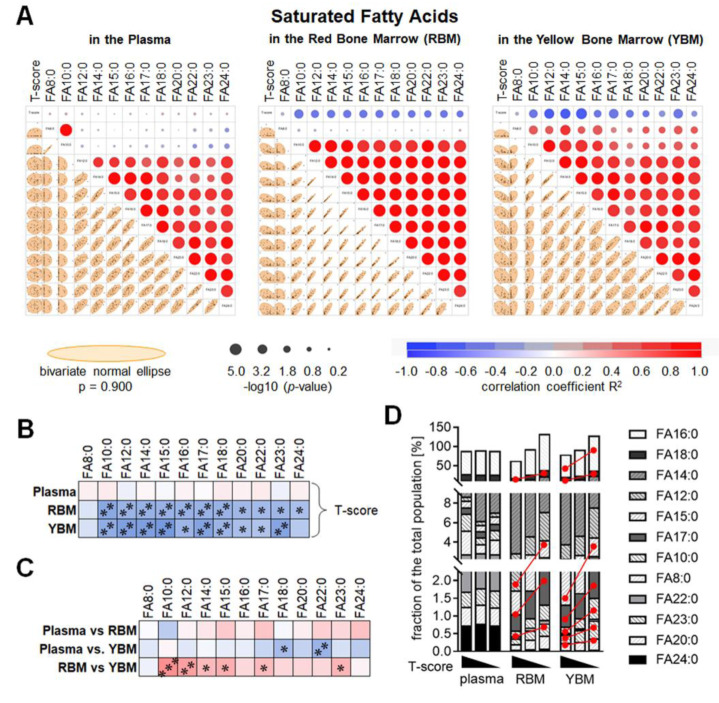
The composition of saturated fatty acids (SAT) in the red bone marrow (RBM) and yellow bone marrow (YBM) is altered depending on the age-adjusted bone mineral density (T-score). (A) Multivariate analysis of the measured SAT in the plasma, RBM, and YBM. Correlations are displayed as bivariate fitted normal ellipses (*p* = 0.90) with individual data points and summarized as colored significance circles. (B) Summary of the correlations of the T-score and the individual SAT in the plasma, RBM, and YBM. (C) Summary of the correlations of the individual SAT measured in plasma, RBM, and YBM. Colors represent the correlation (red: positive / blue: negative) with * *p* < 0.05, ** *p* < 0.01, and *** *p* < 0.001. (D) The composition of the individual SAT based on the T-score in the plasma, RBM, and YBM is summarized as stacked bars. The amount of SAT is presented as % of the average amount of all 30 samples investigated. The red lines mark significant (*p* < 0.05) changes between the three groups (normal bone mineral density / osteopenia / osteoporosis).

The composition of SAT in the plasma, RBM, and YBM had in common that the levels of FA14:0, FA15:0, FA16:0, FA17:0, FA18:0, FA20:0, FA22:0, FA23:0, and FA24:0 showed a strong positive correlation. In the RBM and the YBM this was also valid for FA10:0, and exclusively in the YBM also for FA8:0. In contrast, in the plasma, FA8:0 and FA10:0 showed a strong positive correlation independent of the other SAT measured (Figure 2A(Fig. 2)). None of the SAT measured in the plasma showed a correlation with the T-score. In contrast, almost all SAT measured in the RBM and YBM showed a negative correlation with the T-score. FA8:0 showed no correlation with the T-score (Figure 2B(Fig. 2)). Comparing the levels of the individual SAT in the plasma, RBM, and YBM revealed that there was no correlation between the individual SAT measured in the plasma and RBM, but a weak negative correlation between FA18:0 and FA22:0 measured in the plasma and YBM. In contrast, FA10:0, FA12;0, FA14:0, FA15:0, FA17:0, and FA23:0 measured in the RBM and YBM correlated positively (Figure 2C(Fig. 2)). Comparing the average levels of the SAT within the three groups investigated (normal BMD / osteopenia / osteoporosis) showed significantly increased levels of FA15:0, FA17:0, FA18:0, and FA23:0 in the RBM and YBM of patients with osteoporosis when compared to controls. Additionally, levels of FA10:0, FA16:0 and FA20:0 were significantly increased in the YBM of patients with osteoporosis when compared to controls (Figure 2D(Fig. 2)).

### The amount of the mono-unsaturated fatty acids FA18:1 n-9 and FA20:1 n-9 is elevated in the yellow bone marrow of patients with osteoporosis

**Figure 3:**
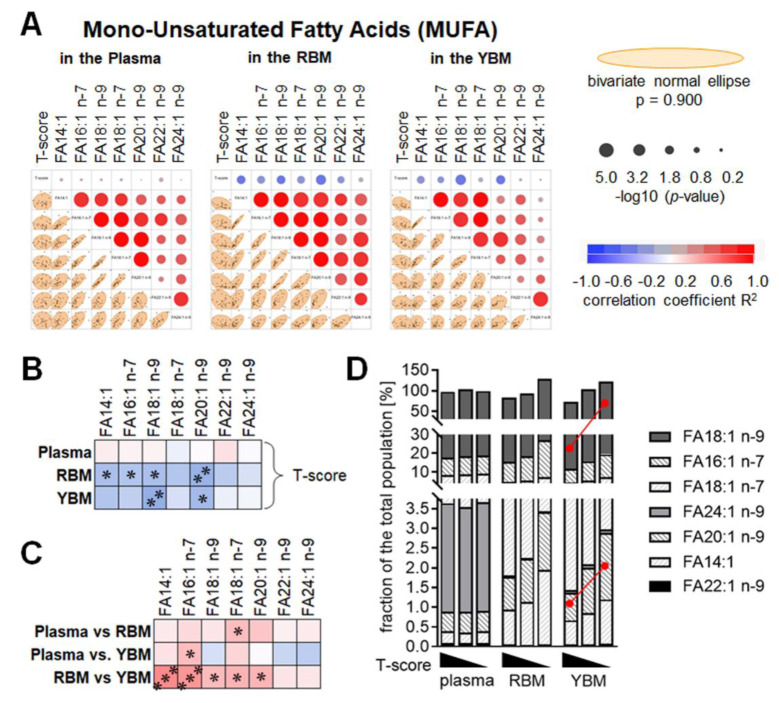
The composition of mono-unsaturated fatty acids (MUFA) is altered depending on the age-adjusted bone mineral density (T-score), especially in the yellow bone marrow (YBM). (A) Multivariate analysis of the measured MUFA in the plasma, red bone marrow (RBM), and YBM. Correlations are presented as bivariate fitted normal ellipses (*p* = 0.90) with individual data points and summarized as colored significance circles. (B) Summary of the correlations of the T-score and the individual MUFA in the plasma, RBM, and YBM. (C) Summary of the correlations of the individual MUFA measured in plasma, RBM, and YBM. Colors represent the correlation (red: positive / blue: negative) with * *p* < 0.05, ** *p* < 0.01, and *** *p* < 0.001. (D) The composition of the individual MUFA based on the T-score in the plasma, RBM, and YBM is summarized as stacked bars. The amount of MUFA is presented as % of the average amount of all 30 samples investigated. The red lines mark significant (*p* < 0.05) changes between the three groups (normal bone mineral density / osteopenia / osteoporosis).

The composition of MUFA in the plasma, RBM, and YBM had in common that all the MUFA measured positively correlated with each other. The strongest correlation was observed in RBM, followed by plasma and YBM (Figure 3A(Fig. 3)). Again, none of the MUFA measured in the plasma showed a correlation with the T-score. The MUFA quantified in the RBM and YBM showed a negative correlation with the T-score, which was most pronounced for FA14:1, FA16:1 N-7, FA18:1 n-9, and FA20:1 n-9 in the RBM and FA18:1 n-9 and FA20:1 n-9 in the YBM (Figure 3B(Fig. 3)). Comparing the levels of the individual MUFA in the plasma, RBM, and YBM showed only a weak positive correlation between FA18:1 n-7 measured in the plasma and RBM, and FA16:1 n-7 measured in the plasma and YBM. In contrast, most MUFA measured in the RBM and YBM correlated positively with each other (Figure 3C(Fig. 3)). Comparing the average levels of the MUFA within the three study groups identified significantly elevated levels of FA18:1 n-9 and FA20:1 n-9 only in the YBM of patients with osteoporosis when compared to controls (Figure 3D(Fig. 3)).

### The amount of poly-unsaturated fatty acids is increased especially in the yellow bone marrow of patients with osteoporosis

**Figure 4:**
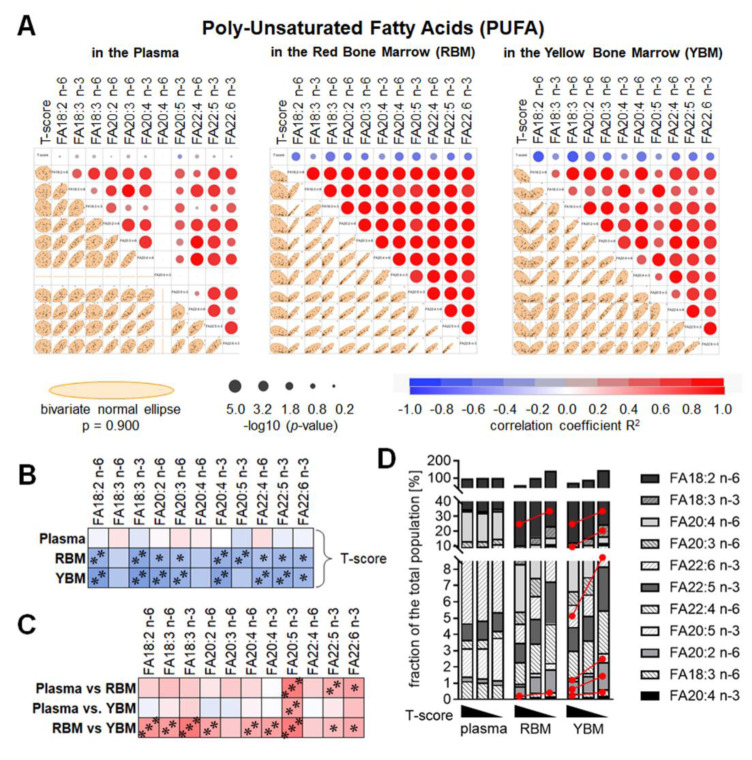
The composition of poly-unsaturated fatty acids (PUFA) in the red bone marrow (RBM) and yellow bone marrow (YBM) is altered depending on the age-adjusted bone mineral density (T-score). (A) Multivariate analysis of the measured PUFA in the plasma, RBM, and YBM. Correlations are presented as bivariate fitted normal ellipses (*p* = 0.90) with individual data points and summarized as colored significance circles. (B) Summary of the correlations of the T-score and the individual PUFA in the plasma, RBM, and YBM. (C) Summary of the correlations of the individual PUFA measured in plasma, RBM, and YBM. Colors represent the correlation (red: positive / blue: negative) with * *p* < 0.05, ** *p* < 0.01, and *** *p* < 0.001. (D) The composition of the individual PUFA based on the T-score in the plasma, RBM, and YBM is summarized as stacked bars. The amount of PUFA is presented as % of the average amount of all 30 samples investigated. The red lines mark significant (*p* < 0.05) changes between the three groups (normal bone mineral density / osteopenia / osteoporosis).

The composition of PUFA in the plasma, RBM, and YBM plasma, RBM, and YBM had in common that all the PUFA measured positively correlated with each other. In contrast to the RBM and YBM, which had overall a lower amount of PUFA than the plasma, FA20:4 n-3 (2) was not detectable in the plasma (Figure 4A(Fig. 4)). Similar to SAT and MUFA, none of the PUFA measured in the plasma showed a correlation with the T-score, but many of the PUFA measured in the RBM and YBM negatively correlated with the T-score (Figure 4B(Fig. 4)). Comparing the levels of the individual PUFA in the plasma, RBM, and YBM revealed positive correlations between FA20:5 n-3, FA22:5 n-3, and FA22:6 n-3 measured in the plasma and RBM, and FA20:5 n-3 measured in the plasma and YBM. In contrast, most PUFA measured in the RBM and YBM correlated positively with each other (Figure 4C(Fig. 4)). Comparing the average levels of the PUFA within the three study groups investigated showed significantly increased levels of FA18:2 n-6 and FA20:4 n-3 in both the RBM and YBM of patients with osteoporosis when compared to controls. In addition, levels of FA18:3 n-3, FA20:2 n-6, FA20:5 n-3, and FA22:6 n-3 were significantly increased in the YBM of patients with osteoporosis when compared to controls (Figure 4D(Fig. 4)).

### TRAP5b levels are negatively associated with T-scores and positively with selected fatty acids in the plasma, red-, and yellow bone marrow

**Figure 5:**
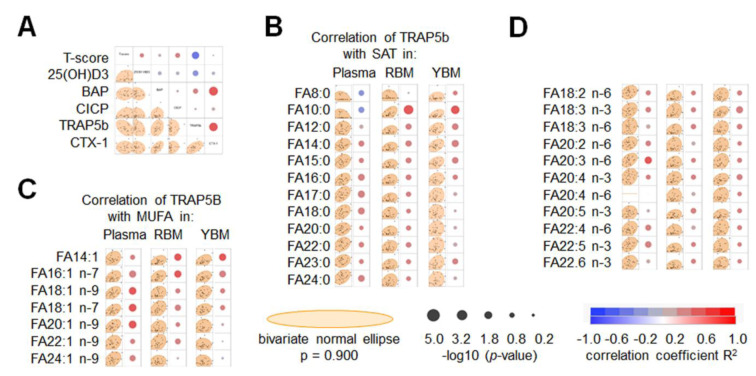
Correlation of the specific bone markers in the plasma with age-adjusted bone mineral density (T-score) and fatty acid composition. (A) Multivariate analysis of the measured bone markers in the plasma and the corresponding T-scores. Summary of the correlations of the TRAP5b levels measured in the plasma with (B) the individual saturated fatty acids (SAT), (C) mono-unsaturated fatty acids (MUFA), and (D) poly-unsaturated fatty acids (PUFA) in the plasma, red bone marrow (RBM), and yellow bone marrow (YBM). Correlations are displayed as bivariate fitted normal ellipses (*p* = 0.90) with individual data points and summarized as colored significance circles.

In order to identify if alterations in the BMD are associated more with a decreased osteoblast activity or an increased osteoclast activity in our study population, levels of bone markers were detected in the plasma. While the osteoblast marker BAP showed no association with the T-scores, the TRAP5b levels showed a significant (*p* = 0.010) negative correlation with the T-scores (Figure 5A(Fig. 5)). Considering the associations between T-scores and the individual fatty acids measured, the next step screened for correlations between the TRAP5b levels and the individual fatty acids measured in the plasma, RBM, and YBM. In contrast to the T-score, TRAP5b levels were associated with several fatty acids measured not only in the RBM and YBM, but also in the plasma. From all SAT measured, FA10:0 showed inverse results in plasma and bone marrow; while showing the strongest positive correlation with TRAP5b in the RBM and YBM (*p* = 0.003 and *p* = 0.008, respectively), TRAP5b levels in the plasma were negatively associated with TRAP5b (*p* = 0.081). Furthermore, FA16:0 (*p* = 0.041), FA17:0 (*p* = 0.029), FA18:0 (*p* = 0.037), and FA24:0 (*p* = 0.044) in the plasma positively correlated with TRAP5b. In contrast, only FA14:0 (*p* = 0.033) in the YBM positively correlated with TRAP5b (Figure 5B(Fig. 5)). All MUFA and PUFA measured in the plasma, RBM, and YBM positively associated with TRAP5b levels. Correlations between MUFA and TRAP5b were significant for FA16:1 n-7 (*p* = 0.031), FA18:1 n-9 (*p* = 0.019), FA18:1 n-7 (*p* = 0.015), and FA20:1 n-9 (*p* = 0.014) measured in the plasma, as well as FA14:1 (*p* = 0.028 and *p* = 0.025) and FA16:1 n-7 (*p* = 0.028 and *p* = 0.043) measured in the RBM and YBM (Figure 5C(Fig. 5)). Correlations between PUFA and TRAP5b were significant only for FA20:3 n-6 (*p* = 0.026) measured in the plasma and FA18:3 n-6 (*p* = 0.034) measured the YBM (Figure 5D(Fig. 5)).

### The fatty acid composition not only in the bone marrow but also in the plasma is well associated with the age-adjusted BMD

**Figure 6:**
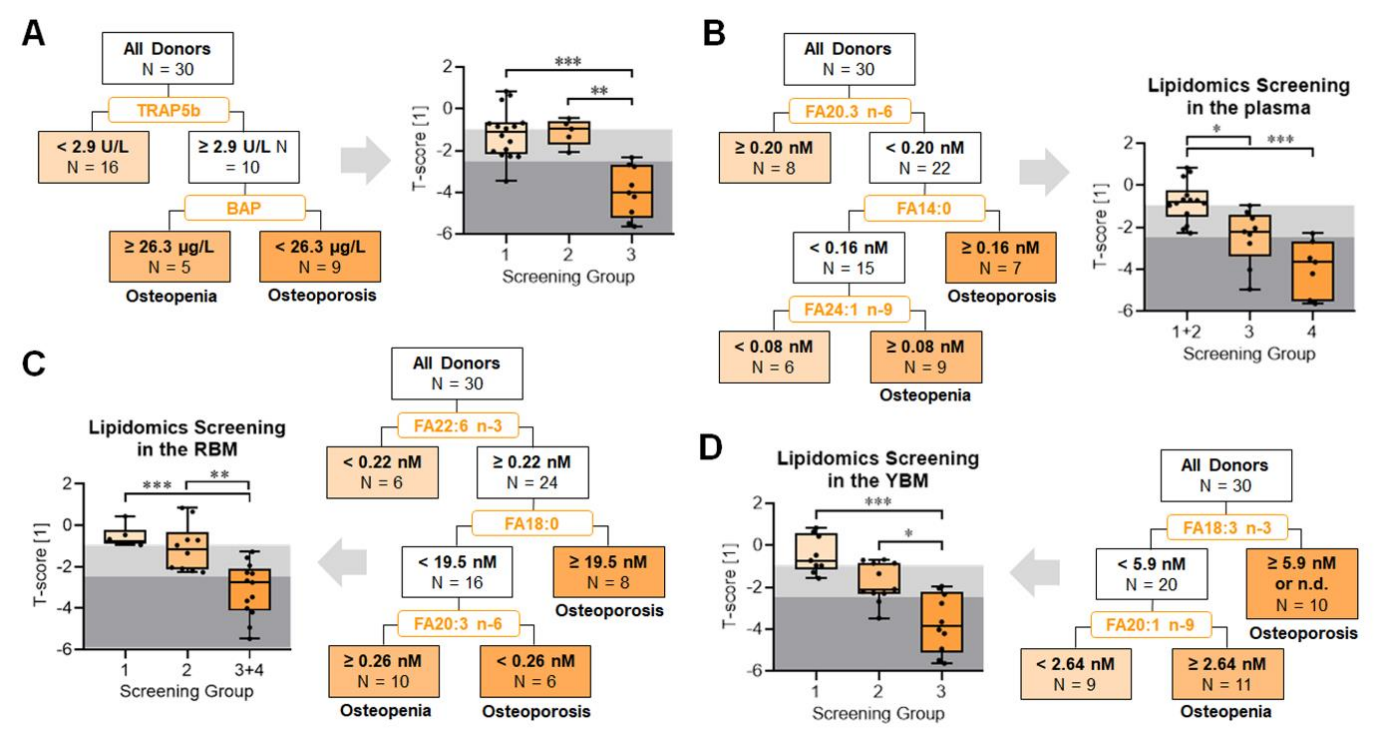
Potential screening strategies for reduced bone mineral density were identified using partition analysis. (A) Screening for reduced age adjusted bone mineral density (T-score) based on the bone markers detected in the plasma. Screening for reduced T-scores based on the fatty acid composition in (B) the plasma, (C) the red bone marrow (RBM), and (D) the yellow bone marrow (YBM). Screening strategies are presented as flow diagrams. Screened datasets are presented as box plots with individual data points. Groups were compared with non-parametric Kruskal-Wallis test, followed by Dunn's multiple comparison test. * *p* < 0.05, ** *p* < 0.01, and *** *p* < 0.001 as indicated.

Plasma levels of BAP and TRAP5b, the established markers for bone turnover, indicated increased bone resorption in our study group with osteoporosis. In this study population, a screening based on these two parameters identified patients with osteoporosis with the highest accuracy (sensitivity: 88.9 %; specificity: 95.2 %; accuracy 93.3 %; *p* < 0.001), however, failed to accurately identify (sensitivity: 57.9 %; specificity: 72.7 %; accuracy 63.3 %; *p* = 0.142) patients with osteopenia and osteoporosis (Figure 6A(Fig. 6)). Although, plasma levels of the measured fatty acids showed no association with the T-scores in our study population, a screening based on these parameters identified patients with osteoporosis with comparably high accuracy (sensitivity: 66.7 %; specificity: 95.2 %; accuracy 86.7 %; *p* < 0.001) than the bone markers. In contrast to the bone markers, this screening could also accurately identify (sensitivity: 75.0 %; specificity: 90.0 %; accuracy 80.0 %; *p* = 0.001) patients with osteopenia and osteoporosis (Figure 6B(Fig. 6)). As the fatty acid composition in the RBM showed most correlations with the T-scores, a screening based on these parameters identified patients with osteoporosis with the highest sensitivity (sensitivity: 100.0 %; specificity: 76.2 %; accuracy 83.3 %; *p* < 0.001). This screening identified patients with osteopenia and osteoporosis not only with the highest sensitivity but also with the highest accuracy (sensitivity: 100.0 %; specificity: 54.5 %; accuracy 83.3 %; *p* < 0.001 / Figure 6C(Fig. 6)). A screening based on the fatty acid composition in the YBM identified patients with osteoporosis with a high accuracy (sensitivity: 77.8 %; specificity: 85.7 %; accuracy 83.3 %; *p* = 0.002), but could not reliably identify (sensitivity: 81.0 %; specificity: 55,6 %; accuracy 73.3 %; *p* = 0.082) patients with both osteopenia and osteoporosis (Figure 6D(Fig. 6)).

## Discussion

Our data show significant associations between the age-adjusted BMD and the fatty acid composition in the RBM and YBM but not in the plasma of our study group. On average the plasma contained approx. 40 % SAT, 27 % MUFA, and 33 % PUFA. Interestingly, the average composition in the bone marrow was different. With 36 % the average amount of SAT was comparable to the plasma but the average amount of MUFA (52 %) and PUFA (12 %) revealed a higher proportion of MUFA than PUFA in the bone marrow. In line with the literature (Cohen et al., 2012[4]; Devlin and Rosen, 2015[6]), the amount of fatty acids increased with decreasing T-score, especially in the YBM - which in the osteoporotic group contained almost double the amount of fatty acids than in the control group. Contrary to earlier assumptions that marrow adipocytes are mainly quiescent and metabolically inert, recent studies have revealed that MAT may be highly metabolically active, responsive to physiological stimuli, and actively interacting with osteoblasts and osteoclasts to mediate bone homeostasis (Sheu and Cauley, 2011[29]; Krings et al., 2012[16]). The fatty acid composition may be the key factor in this process. For example, anti-inflammatory omega-3 PUFA may counteract the effects from pro-inflammatory SAT in developing calcified plaques in atherosclerosis, however, their effect on BMD is less clear.

Studies quantifying the general amount and composition of MAT with non-invasive imaging techniques, *e.g.* proton magnetic resonance spectroscopy (Schellinger et al., 2004[27]; Yeung et al., 2005[31]) or chemical shift encoding-based water-fat magnetic resonance imaging (Beekman et al., 2022[2]), uniformly suggested that an increase in SAT is mainly responsible for the increase in MAT in osteoporosis. However, quantifying the individual fatty acid composition in the bone marrow with gas chromatography could not confirm this result (Griffith et al., 2009[10]; Miranda et al., 2016[21]). In the first study no significant alterations in the fatty acid composition could be observed, which was partly explained by the different donor sites the bone marrow was obtained from (Griffith et al., 2009[10]). Such a donor site variation was excluded in our study, where all samples were obtained from the femur head. Similarly, in the second study where solely iliac crest biopsies from postmenopausal women were compared. In these samples, decreasing BMD was associated with decreased levels of SAT and increased levels of MUFA, especially in those patients which had experienced fragility fractures (Miranda et al., 2016[21]). However, the comparability of these studies is limited - besides a possible influence of the donor site (Griffith et al., 2009[10]), the fatty acid composition in these studies is described as molar percentages of the total fatty acid profile, which was different in each study. Especially, the amount of the long chain SAT, *e.g.* FA18:0, FA20:0 and FA22:0, seemed to be significantly affected by the donor site (Griffith et al., 2009[10]). In our samples, significantly elevated levels of the SAT FA10:0, FA15:0, FA16:0, FA17:0, FA18:0, FA20:0, and FA23:0 were detected in especially the YBM of patients with osteoporosis when compared to controls. Of these plasma levels of FA16:0, FA17:0, and FA18:0 were positively associated with the osteoclast activity in these patients, as measured by circulating TRAP5b levels. This finding is supported by a study showing enhanced osteoclastogenesis in mice on a high-fat diet enriched with FA16:0 (palmitic acid). A SAT induced inflammatory reaction with induction of TNF-α, was identified as key regulator in this process (Drosatos-Tampakaki et al., 2014[7]). In the bone marrow, however, there was a strong association of FA10:0 with the circulating TRAP5b levels, suggesting a positive effect on osteoclast formation or function. Unfortunately, FA10:0 (capric acid) was not measured in the two aforementioned studies (Griffith et al., 2009[10]; Miranda et al., 2016[21]). However, our results were supported by a mouse model investigating the effect of medium‑chain triglyceride ketogenic diet on bone mineral density. Dietary supplementation with capric acid, and even more with caprylic acid (FA8:0), resulted in decreased BMD and increased circulating TRAP5b levels (Jain et al., 2021[13]). In contrast, capric acid was reported to effectively inhibit osteoclast formation *in vitro *(Park et al., 2011[24]; Kim et al., 2014[15]).

In the iliac crest biopsies of postmenopausal women a significant increase in MUFA was observed especially in those samples with osteoporosis and a history of fragility fractures (Miranda et al., 2016[21]). In the present study the increase in the amount of MUFA was less pronounced, solely FA18:1 n-9 and FA20:1 n-9 were significantly increased in the YBM in the osteoporotic group when compared to the control group. In the plasma there was again a positive association of the circulating TRAP5b levels with FA18:1 n-9 (oleic acid), FA18:1 n-7 (vaccenic acid), and FA20:1 n-9 (eicosenoic acid), again suggesting a stimulatory effect on osteoclasts. Of particular interest is the highly specific trans fatty acid vaccenic acid, whose consumption in dairy products has been associated with the incidence of hip fractures in different European countries. As potential mechanism inhibition of alkaline phosphatase activity and resulting calcification has been proposed. Interestingly, dietary supplementation with oleic acid could partly counteract this effect (Hamazaki et al., 2016[11]). In another study a positive effect of oleic acid on BMD was explained with an increase in OPG/RANKL ratio, suppressing osteoclastogenesis (Martin-Bautista et al., 2010[19]), however, in this study diary supplementation was not oleic acid alone, but contained also fish oil (omega-3 PUFA) and vitamins. Likewise, dietary supplementation with oleic acid reduced saturated fatty acid-induced osteoclastogenesis in a mouse model (Drosatos-Tampakaki et al., 2014[7]). However, the triglyceride accumulation favored by oleic acid might further expand the MAT. This finding is further supported by two *in vitro* studies, showing enhanced triglyceride accumulation in oleic acid treated osteoprogenitor cells. While in the first study oleic acid also improved osteogenic function (alkaline phosphatase activity) in the murine bone marrow-derived stroma cell line ST2 (Deshimaru et al., 2005[5]), the opposite was observed for murine MC3T3-E1 osteoprogenitor cells in the second study (Hutchins et al., 2011[12]). In yet another study it was proposed that melatonin could counteract fatty-acid induced triglyceride accumulation in rat ROS17/2.8 osteoprogenitor cells (Sanchez-Hidalgo et al., 2007[26]), which is of potential interest for clinical application.

As mentioned before, in the current study, the overall amount of PUFA increased the most with decreasing BMD. When comparing the osteoporotic group with the control group, especially, FA18:2 n-6, FA18:3 n-3, FA20:2 n-6, FA20:4 n-3, FA20:5 n-3, and FA22:6 n-6 were significantly increased in the YBM. Increased levels of FA18:2 n-6 (linoleic acid) have also been found in the osteoporotoc iliac crest biopsies from postmenopausal women. In contrast, in these samples FA20:3 n-6 and FA20:4 n-6 levels were significantly decreased, especially when donors had a history of fragility fractures (Miranda et al., 2016[21]). Interestingly, the same PUFA (FA20:3 n-6) measured in the plasma showed a strong positive correlation with circulating TRAP5b levels in the present study. As the omega-6 (n-6) PUFA represent precursors to the most strongly pro-inflammatory eicosanoids, an induction of osteoclastogenesis *via* TNF-α (as reported above (Drosatos-Tampakaki et al., 2014[7])) could be considered. This assumption is supported by the study reporting increased levels of omega-6 (n-6) PUFA in MAT derived from femoral heads of patients with osteoarthritis when compared to patients with osteoporosis (Plumb and Aspden, 2004[25]). A diet rich in anti-inflammatory omega-3 (n-3) PUFA has been reported to improve BMD in different rodent models - for review see (Sharma and Mandal, 2020[28]). In the present study, certain anti-inflammatory omega-3 (n-3) PUFA have been increased in bone marrow. For example, α-linoleic acid (FA18:3 n-3) in the YBM not only negatively associated with T-scores, but also positively with circulating TRAP5b levels, suggesting active bone resorption. This is in contrast to a study in rats, which reported that α-linolenic acid reduced bone loss caused by a high-fat diet. In this study, α-linoleic acid had no effect on osteoclast function but stimulated osteoblast function (Chen et al., 2019[3]). In the present study, no significant association between osteoblast activity, measured by BAP levels, and any of the SAT, MUFA, or PUFA could be detected. *In vitro*, medium supplementation with PUFA FA20:5 n-3 and FA22:6 n-3 even suppressed alkaline phosphatase activity in MC3T3-E1 cells (Hutchins et al., 2011[12]). Interestingly, dietary uptake of these two PUFA were positively associated with BMD in a large female cohort investigated in Spain (Lavado-Garcia et al., 2018[17]). Overall, however, evidence is still low that dietary supplementation with omega-3 (n-3) PUFA has a real beneficial effect on BMD in human studies - for review see (Orchard et al., 2012[23]).

## Conclusion

In this study, the observed alterations in the fatty acid composition contained specific SAT, MUFA, and PUFA, which in combination gave a characteristic pattern for patients with normal BMD, osteopenia, and osteoporosis. The selective markers for their differentiation differ between plasma, RBM, and YBM and partly correlated with osteoclast activity, suggesting a possible mechanism how these fatty acids may interfere with BMD. The observation that there was no general correlation between the T-scores and the plasma levels of the measured fatty acids, however, a screening based on these parameters reliably identified patients with osteopenia and osteoporosis, suggests that there is not a single fatty acid that can be claimed for controlling BMD. The overall, positive effects of omega-3 (n3) fatty acids on BMD observed in rodents studies, could be explained by the genetic homogeneity of the animals (Jiao et al., 1990[14]). The genetic variability in humans should be considered, especially, when dietary supplementation with fatty acids is attempted (Garcia-Rios et al., 2012[9]).

## Notes

Anna J. Schreiner and Claudine Seeliger contributed equally as second author.

## Figures and Tables

**Table 1:**
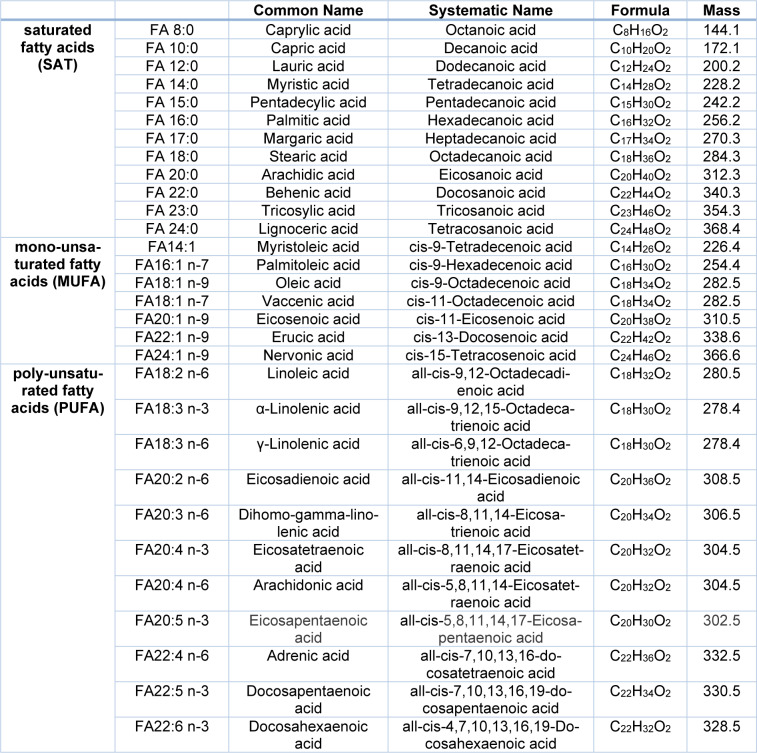
Overview of the measured fatty acids
